# Cross-Cultural Psychometric Analysis of the Mature Happiness Scale-Revised: Mature Happiness, Psychological Inflexibility, and the PERMA Model

**DOI:** 10.1007/s10902-023-00633-7

**Published:** 2023-02-16

**Authors:** David F. Carreno, Nikolett Eisenbeck, James Greville, Paul T. P. Wong

**Affiliations:** 1 Department of Psychology, University of Almeria, Carr. Sacramento, s/n, 04120 La Cañada, Almería Spain; 2 Department of Personality, Evaluation and Psychological Treatment, Faculty of Psychology, University of Seville, Seville, Spain; 3 University of South Wales, Newport, UK; 4 Meaning-Centered Counselling Institute Inc., North York, ON Canada

**Keywords:** Mature Happiness Scale, Mature happiness, Inner harmony, PERMA, Psychological inflexibility, Validation

## Abstract

The present study aimed to evaluate the psychometric properties of the Mature Happiness Scale, a measure focused on inner harmony. Mature happiness is achieved when a person can live in balance between both positive and negative aspects of their life. A total sample of 2,130 participants from five countries (Canada: *n* = 390, United States: *n* = 223, United Kingdom: *n* = 512, Spain: *n* = 724, and Hungary: *n* = 281) responded to an online survey including the original Mature Happiness Scale, the PERMA-Profiler, and the Acceptance and Action Questionnaire-II. Exploratory and confirmatory factor analyses yielded a one-factor solution with seven positive items (non-reversed). We called this new version of the questionnaire the Mature Happiness Scale-Revised (MHS-R). Measurement invariance was found across countries, age groups, gender, and mental disorder diagnosis. Internal consistency and test–retest reliability were high. Older people, males, and people without a mental disorder diagnosis scored higher in mature happiness than younger ones, females, and those with a mental health disorder diagnosis, respectively. Mature happiness showed strong positive associations with various subscales of the PERMA-Profiler, specifically with positive emotions and meaning in life. In addition, mature happiness was strongly correlated with less negative affect and inner conflict and lower psychological inflexibility, whereas it was moderately correlated with lower loneliness. This validity evidence supports the cross-cultural use of the MHS-R in the aforementioned countries to reliably measure happiness among adults. With its holistic approach, the MHS-R may be a unique complement to other well-being measures, particularly to better predict mental health problems.

## Introduction

The construct of well-being has evolved from different theoretical and philosophical approaches (e.g., Diener, 1984; Keyes, 2002; Ryff, 1989, 2014; Seligman, 2011). Among the challenges for traditional conceptualizations of well-being is the inclusion of negative emotions. The relationship between the so-called positive and negative emotions is much more complex than simply being opposite ends of a spectrum (Diener & Emmons, 1984; Watson et al., 1988; Zevon & Tellegen, 1982). Given their complex relationship, positive affect and negative affect have been considered two separate constructs to be assessed in traditional measures of well-being (Bradburn, 1969; Butler & Kern, 2016; Diener et al., 1985; Watson et al., 1988). However, most of these well-being approaches such as the PERMA model (acronym established by Seligman, 2011, to refer to a well-being model based on Positive emotions, Engagement, Relationships, Meaning, and Achievement) do not incorporate negative emotions *into* the conceptualization of well-being. One of the main critiques of the *first wave of positive psychology* (Seligman & Csikszentmihalyi, 2000) has been this neglection of negative emotions, framing them as if they simply represent the absence of positive states (e.g., Held, 2004; Wong & Roy, 2017).

### Mature Happiness: A Holistic Approach to Well-Being Based on Inner Harmony

An alternative approach to well-being has been formalized in *existential positive psychology* (PP2.0, Wong, 2009), also called the *second wave of positive psychology* (Ivtzan et al., 2015; Lomas & Ivtzan, 2016). PP2.0 may be distinguished from the first wave of positive psychology (Seligman & Csikszentmihalyi, 2000) because it highlights the importance of negative emotions and stressful events in the understanding of well-being. As noted by Lomas et al. (2021), from the second wave of positive psychology perspective, there is a difference between positive and negative *valence* (whether something is experienced as pleasant or unpleasant) and positive and negative *outcome* (whether something facilitates or hinders well-being). PP2.0 emphasizes that, while adverse experiences and suffering may intuitively be seen as undesirable parts of life, they are not only inevitable but can indeed serve as promoters of personal growth and resilience (Calhoun & Tedeschi, 2006; Ivtzan et al., 2015; Kashdan & Biswas-Diener, 2014). Negative emotions have of course played an adaptive, protective role in human evolution (see Nesse, 2019). In this vein, several studies have shown the mental health benefits of accepting painful emotions and thoughts (e.g., Levin et al., 2012; Stockton et al., 2019), meanwhile rigid patterns of avoiding unpleasantness or difficulty have been linked to psychological problems (e.g., Chawla & Ostafin, 2007; Spinhoven et al., 2014). These findings suggest that the relationship one has with suffering should be integrated into the conceptualization of well-being and happiness.

In one of the recent efforts to do so, Wong and Bowers (2018) proposed an integrative concept of well-being called *mature happiness*. This construct refers to a positive state of mind characterized by inner harmony, calmness, acceptance, contentment, and satisfaction with life (Wong & Bowers, 2018). Mature happiness is understood as the positive mental state resulting from living in balance with the various aspects of one’s life, including both positive and negative. Rooted in the principles of Confucianism, Dao/Taoism, and Buddhism, mature happiness addresses the degree to which a person lives in harmony with their strengths and weaknesses, with other people, and with the world in general (Haybron, 2013; Wong, 2014). It is called “mature” because this type of well-being requires a significant level of personal maturity, particularly to live in attunement with one’s vulnerabilities (accepting painful emotions, personal burdens, and the fragility of human existence).

This concept of happiness thus goes beyond subjective/hedonic happiness and eudaimonic happiness, by including a third type of happiness, called *chaironic* happiness (Wong, 2011). Chaironic happiness (from Greek: “chairo” meaning “blessing” or “joy”) is related to harmony, the adoption of a positive mature attitude towards life, being mindful of the present moment, and being attuned to a self-transcendental existence (Wong, 2011; for similar considerations see Aydin & Khan, 2021). The positive state of mature happiness differs from the positive emotions included in the traditional approaches to subjective well-being such as joy, contentment, excitement, or enthusiasm (Diener et al., 1985; Watson et al., 1988) as it represents the *overall* positive state resulting from being in attunement with *both* positive and negative aspects of one’s life. In contrast, the positive emotions included in subjective well-being are related to fluctuating happiness, which normally is associated with higher arousal and less stability, and may not be that dependent on how one relates to their negative side. As a result, mature happiness is considered durable happiness because of its higher stability, low-arousal feelings, and relationship with self-transcendence (Wong et al., 2021). This difference between fluctuating happiness and durable happiness has been extensively reviewed by Dambrun and Ricard (2011). Mature happiness fits into the category of the so-called *authentic-durable happiness*. Apart from the previously mentioned aspects, authentic-durable happiness is characterized by selflessness and self-transcendence, while hedonic-fluctuating happiness is related to self-centeredness and self-enhancement (Dambrun et al., 2012).

Although the research on harmony in psychology has been quite scattered and disconnected, the conceptualization of well-being based on harmony has gained considerable support in recent years (for a review, see Lomas, 2021). For example, Delle Fave et al. (2016) reported that inner harmony and relational connectedness are the most-mentioned aspects that laypeople associate with happiness. Definitions of inner harmony included terms such as inner peace, emotional stability, balance, acceptance of life, serenity, and contentment. Satisfaction (with life and oneself), positive emotions (joy, cheerfulness, vitality, enthusiasm, and elation), and health were also mentioned as facets of happiness but to a lesser degree.

Mature happiness can be considered one of the experiential outcomes of living a meaningful life (Wong, 2020). This construct is related to the stoic concept of *apatheia*, which describes a state of peace of mind and equanimity in the face of passions (a term in the stoic literature that refers to disturbing emotions such as distress, fear, lust, and excessive delight). Apatheia has been considered to be a principal constituent of the eudaimonic life (Pigliucci, 2020). Despite the connection between eudaimonic well-being and mature happiness, an important distinction should be made; while eudaimonic happiness is focused on the pursuit of virtue, meaning, and flourishing; mature happiness aims to achieve equanimity and peace of mind, being mindful of and attuned to one’s nature as a human being, and finding a balance between the dialectics of life (Wong, 2011). From this theoretical perspective, mature happiness may represent a relevant facet of well-being to be taken into account, particularly in circumstances in which people are amid adversity (i.e. achievement and pursuit of excellence is difficult), such as during the challenges of the global COVID-19 pandemic. An appropriate measure of mature happiness may therefore be an invaluable tool for research on suffering vs. flourishing, particularly during this unique period in our collective history.

### The Mature Happiness Scale and Other Related Measures

Thus far there is only one scale aimed to measure mature happiness, called the Mature Happiness Scale (MHS, Wong & Bowers, 2018). This instrument has been used in recent studies (e.g., Carreno et al., 2021; Wong et al., 2021). For instance, in a study during the initial phase of the COVID-19 pandemic, Carreno et al. (2021) observed that mature happiness appeared as a related but separate factor from PERMA (latent correlation of 0.78). In this study, a revised version of the MHS, which contains exactly the same items as the scale presented in the current paper, predicted psychological distress beyond and above the PERMA-Profiler. Mature happiness was a better negative predictor of stress, anxiety, and general distress, while the PERMA-Profiler showed to be a better negative predictor of depression. Additionally, mature happiness acted as a moderator between the perceived noxious effects of the pandemic and all markers of distress (stress, anxiety, depression, and general distress). At the same time, the PERMA-Profiler moderated the relationship between stress and depression. The revised version of the MHS used by Carreno et al. (2021) was the result of an initial factorial analysis, however, the authors did not provide any detailed information about this and other steps of the scale validation process. Most of the MHS psychometric properties are yet to be analyzed and published. This was the main purpose of the present study.

In the literature, we have identified at least other three existing measures of harmony. One is the Peace of Mind Scale (Lee et al., 2013) which assesses the affective well-being valued in Chinese culture. This measure includes feelings such as peace and stability (e.g., “I have peace and harmony in my mind”). The second related measure is the Harmony in Life Scale (Kjell et al., 2016) which assesses the subjective perception of overall harmony in life. According to the conceptualization of this scale (Kjell et al., 2016), harmony is characterized by selflessness, relatedness, mindfulness, psychological flexibility, sustainability, and secondary control, that is, it represents how an individual aligns or adapts to their environment (Kjell, 2011). This construct is different from the construct of satisfaction with life, which is characterized by self-centeredness, self-enhancement, fluctuation, and primary control; it addresses an individual’s ability to change their environment according to their wishes and needs. Thus, the Harmony in Life Scale measures cognitive evaluations of one’s relationship with life as a whole (e.g., “Most aspects of my life are in balance”, “I fit in well with my surroundings”). The third measure similar to the MHS is the Subjective Authentic-Durable Happiness Scale (Dambrun et al., 2012), which was developed to compare fluctuating happiness and authentic-durable happiness.

In contrast with the MHS however, these three scales focus on *either* feelings of peace *or* harmony with life (in terms of fitting into the personal context). Furthermore, these scales do not explicitly address negative aspects of living. The MHS meanwhile combines *both* feelings of peace and harmony with life (e.g., “I feel comfortable in my own skin”, “I am able to live in harmony with people that matter to me”), including the *acceptance* of negative aspects of living (e.g., “I have learned to accept life as it is”; “I have learned to let go of all my cares and burdens”). Similar to the MHS, the recently developed Equanimity Scale (Juneau et al., 2020) measures equanimity (even-minded state of mind and hedonic independence), however, this instrument does not represent a measure of happiness since it does not include other positive features such as satisfaction with life and contentment. For the aforementioned reasons, the MHS may collect important features in the evaluation of happiness and inner harmony beyond other measures in the literature.

### Psychological Inflexibility and Mature Happiness

On the other side of the spectrum, the opposite of mature happiness can be understood as a mental state in which the person feels broken, experiencing an inner battle with their emotions and their life as a whole. This negative state is often linked to feelings of being disconnected from oneself, others, and the world. Some of the proposed items in the original MHS are aimed to measure this state (e.g., “I am troubled by inner conflict”, “My conscience bothers me”). In this state of mind, the person usually attempts to avoid their painful emotions and thoughts at all costs because they have acquired strong aversive functions. Thus, it is very unlikely that the person would accept these private events and would be willing to be in contact with them. This approach strongly resembles the construct of *psychological inflexibility*: the rigid avoidance of negative emotions and thoughts that is incompatible with actions based on personal values (Bond et al., 2011). Not surprisingly, several studies have demonstrated the links between psychological inflexibility and mental health problems (Bond et al., 2011; Kashdan & Rottenberg, 2010; Levin et al., 2014).

Instead, the opposite of psychological inflexibility, termed *psychological flexibility*, is defined as “the ability to contact the present moment more fully as a conscious human being, and to either change or persist when doing so serves valued ends” (Hayes et al., 2006). This construct is related to the ability to accept unpleasant emotions and thoughts and act in accordance with personal values, and it has been related to positive mental health outcomes (Kashdan & Rottenberg, 2010).

Of note, the construct of psychological flexibility, because of its behavioral background, is defined in terms of the ability to respond to one’s emotions and thoughts. Therefore, this construct is based on *cognitive-behavioral processes* (Hayes et al., 2006; Hofmann & Hayes, 2019). However, the conceptualization and the evaluation of psychological flexibility do not include the *experiential/emotional state* (e.g., peace of mind, inner harmony, satisfaction with life) resulting from developing such processes. In contrast, the concept of mature happiness is based on this positive state, capturing the emotional experience of acceptance and living in harmony with most parts of oneself and the context in which one lives. Despite these theoretical connections between the two constructs, no previous study has empirically explored their relationship, which constitutes the second aim of this study.

### The Present Study

This study aimed to investigate for the first time the psychometric properties of the MHS (factor structure, measurement invariance, internal consistency, and validity evidence based on the relationship with other measures). In line with positive cross-cultural psychology (Lomas, 2015) and the third wave of positive psychology (Lomas et al., 2021) highlighting the inclusion of multiculturality in positive psychology research, a cross-cultural analysis was performed including participants from five different countries (Canada, United States, United Kingdom, Spain, and Hungary). For the evaluation of concurrent and discriminant validity, we analyzed the relationship of the MHS with the PERMA-Profiler (Butler & Kern, 2016) and the Acceptance and Action Questionnaire-II (AAQ-II; Bond et al., 2011), the latter being a measure of psychological inflexibility. The hypotheses tested to evaluate concurrent and discriminant validity were:

#### Hypothesis 1

Mature happiness would be higher in older people as it requires emotional maturity, which normally increases across the lifespan (Lawton et al., 1992; Márquez-González et al., 2008).

#### Hypothesis 2

Although mature happiness and PERMA represent different constructs (Carreno et al., 2021), MHS scores would be strongly associated (*r* above 0.50) with well-being scores of the PERMA-Profiler (positive emotions, engagement, relationships, meaning, and accomplishment) as both instruments assess subjective and eudaimonic aspects of well-being.

#### Hypothesis 3

Mature happiness would present negative moderate relationships (*r* from − 0.30 to − 0.49) with mental health problems as measured by the PERMA subscale of negative affect and the previous diagnosis of a mental disorder.

#### Hypothesis 4

Mature happiness would show a negative strong association (*r* above − 0.50) with psychological inflexibility since the latter refers to rigid avoidance of negative emotions and thoughts while mature happiness is about accepting and integrating most aspects of oneself.

## Method

### Participants and Procedure

A sample of 2130 people from five different countries participated in the present study (Canada: *n* = 390, United States: *n* = 223, United Kingdom: *n* = 512, Spain: *n* = 724, and Hungary: *n* = 281). This sample was selected from a larger research project (Carreno et al., 2021; Eisenbeck et al., 2022; link to the project: https://www.researchgate.net/project/Psychological-coping-with-the-COVID-19-pandemic). The entry criterion was to be older than 18 years old. The average age of the participants was 38.88 years (*SD* = 13.76), ranging between 18 and 84. The majority of the participants were female (77.4%) and most had no diagnosed mental health condition (83.3%). Detailed sociodemographic characteristics of the sample in each country are provided in Table 1.

**Table 1:** Socio-demographic characteristics of the sample

	Canada	United States	United Kingdom	Spain	Hungary	Total
*n*	390	223	512	724	281	2130
*Age*						
*M (SD)*	36.92 (13.23)	43.70 (15.64)	42.37 (15.26)	36.55 (11.83)	37.40 (12.40)	38.88
Range	18–84	18–77	18–76	18–73	18–71	18–84
Gender (female %)	52.8	81.6	88.1	77.1	89.3	77.4
Mental health diagnosis (no %)	84.6	73.1	74.2	84.4	92.9	88.3
Religiousness (yes %)	55.9	70.4	42.8	41.7	64.1	50.5
*Marital status (%)*						
Single/divorced/widowed	32.6	33.2	36.6	34.9	35.2	34.7
In a relationship	12.3	12.1	13.9	20.0	14.9	15.6
Married/cohabiting with partner	55.1	54.7	47.9	45.0	49.8	49.2
*Highest level of education (%)*						
Primary/secondary	15.1	13.9	28.9	14.9	22.4	19.2
Higher education	74.9	71.3	48.8	72.7	70.1	66.9
Still a student	10.0	14.8	22.3	12.4	7.5	13.9

As a part of a larger project, in this study participants completed various questionnaires about psychological distress and different coping strategies before participating in this part of the project (Eisenbeck et al., 2022). The questionnaires of this study were the last ones of the package (order: PERMA, AAQ-II, and finally, MHS).

The participating investigators agreed on the theoretical framework and the objectives of the study. All translations were made based on the recommended best practices (Beaton et al., 2000), including various translators and back-translations. Data collection methods were identical in all participating countries (for the importance of establishing as much equivalence as possible in cross-cultural research, see for instance Matsumoto & Yoo, 2006). The study was approved by institutional ethics boards for all participating countries.

Participants were recruited through email invitations and social media announcements using snowball sampling. Participation consisted of responding to an online survey that took approximately 15 min. It was anonymous, voluntary, and without any monetary compensation except for part of the Canadian sample that we recruited via MTurk. Participants reported their socio-demographic characteristics (age, gender, mental health diagnosis, religiousness, marital status, economic status, and highest level of education) and completed the MHS, the PERMA-Profiler, and the AAQ-II. Respondents were given the option to leave their email addresses to partake in the second part of the study to evaluate the test–retest reliability of the questionnaire. This measurement took place 3 to 4 weeks after the first one.

### Measures

#### Mature Happiness Scale

The MHS measures different aspects of mature happiness such as inner harmony, calmness, acceptance, contentment, and satisfaction with life (Wong & Bowers, 2018). Participants rate items on a Likert scale from 1 (*not at all*) to 5 (*all of the time*). The original scale contains 12 items with three reversed items that measure inner conflict. However, after the analyses reported in this paper, the final scale, which we called the Mature Happiness Scale-Revised (MHS-R), was composed of seven items without reversed statements (see Appendix). In this analysis, two of the original three reversed items (“I am troubled by inner conflict”, “My conscience bothers me”) appeared as a separate factor from mature happiness (termed “*inner conflict”*, see Results). Total scores of the MHS-R oscillate between 7 and 35, with higher scores indicating higher levels of mature happiness. Correlations between the two items of inner conflict ranged between 0.433 and 0.497 in all countries,* p* > 0.001.

#### PERMA-Profiler

The PERMA-Profiler (Butler & Kern, 2016) measures the five domains of the PERMA model of flourishing: Positive emotion, Engagement, Relationships, Meaning, and Accomplishment. It contains three items for each domain, with the total score representing a global measure of well-being. Additionally, we included the subscales of physical health (three items), negative emotion (three items), and loneliness (one item) proposed by the authors (Butler & Kern, 2016). To be consistent with the rest of the questionnaires, in this study, participants rated on a Likert scale from 0 to 6 instead of 0–10 (as in the original) and questionnaire means were calculated (the sum of all items of each subscale) (see Dawes, 2008). In the participating five countries, Cronbach’s alphas for well-being, physical health, and negative emotion ranged between 0.73 and 0.94.

#### Acceptance and Action Questionnaire-II

The Acceptance and Action Questionnaire-II (AAQ-II; Bond et al., 2011) consists of seven items that measure psychological inflexibility. Respondents rate each statement on a 7-point scale from 1 (*never true*) to 7 (*always true*). Higher scores indicate higher psychological inflexibility, that is, a higher tendency to experiential avoidance that is incompatible with personal values. This questionnaire has shown satisfactory psychometric properties in English (Bond et al., 2011), Spanish (Ruiz et al., 2013), and Hungarian (Eisenbeck & Szabó-Bartha, 2018). Alphas in this study ranged between 0.91 and 0.93.

### Data Analytic Strategy

Data were analyzed using Mplus (Muthén & Muthén, 2016) and SPSS (Version 25). Due to a technical issue, basic demographic data (age, gender) of 40 participants were not recorded and these cases were discarded. Data were excluded in the case of participants (*n* = 11) who failed to complete at least 50% of any of the measures of the study or showed straightlining (e.g., employed the same to all items of a questionnaire even though the items aim to measure in opposite directions, *n* = 12). Missing data were then less than 0.01%, missing completely at random, χ2 (43) = 56.43, *p* = 0.082, and were not replaced. The final sample consisted of 2130 participants. Distribution of the variables was assessed with evaluation of kurtosis, and skewness, and with the Kolmogorov–Smirnov and Shapiro–Wilk tests.

Following the best practices, to evaluate the factor structure of the MHS, we employed exploratory factor analysis (EFA) using principal component analysis with oblimin rotation on the first randomly selected half of the Canadian sample (*n* = 201). Subsequently, confirmatory factor analyses (CFAs) were used on the second random half of the Canadian sample (*n* = 189) and on the rest of the samples to assess the proposed factor structure of the scale (see for instance Clark & Watson, 2019; Fenn et al., 2020; Rosellini & Brown, 2021). To be sensitive to the possible influence of culture to the measures and conclusions (e.g., Chen, 2008), the samples of the five participating countries were treated separately regardless of the language of the country. People can give slightly different meanings to different items based on their culture, which eventually can affect their view of that item and the underlying factor structure of the measure. All analyses were performed using the robust maximum likelihood (MLR) estimator because violations of univariate and multivariate normality were observed. In all cases, sample sizes were adequate for factor analyses: ratios of the number of cases to the number of estimated parameters were above the established threshold of 20 (see Jackson, 2003).

Due to the χ2 test’s sensitivity to sample size (e.g., Hu & Bentler, 1998), model fits were assessed using the Comparative Fit Index (CFI), the Tucker–Lewis index (TLI), the Root-Mean-Square Error of Approximation (RMSEA), and the Standardized Root-Mean-Square Residual (SRMR). Values of CFI and TLI are considered acceptable above 0.90 and excellent above 0.95 (Hu & Bentler, 1998), while SRMR values below 0.80 indicate a good fit (Schreiber et al., 2006). An RMSEA value lower than 0.08 shows a reasonable fit, between 0.08 and 0.10 a mediocre fit, and above 0.10 is unacceptable (Browne & Cudeck, 1993).

Configural, measurement, and scalar invariance were evaluated across countries (Canada, United States, United Kingdom, Spain, and Hungary), age groups (cut-off points for three approximately equal groups were: 18–30, 30–45, and 45 +), gender (male/female), and mental disorder diagnosis (yes/no) in the entire sample. Successively restrictive models were compared with the Satorra–Bentler scaled χ2 difference tests using scaling correction factors and with CFI, RMSEA, and SRMR difference scores, the latter serving as indices for final decisions. As the sample size was large, changes of ≤  − 0.010 for CFI, ≤ 0.015 for RMSEA, and ≤ 0.030 / ≤ 0.010 for SRMR (depending on the level of testing) showed invariance (Chen, 2007).

Descriptive statistics (scale/questionnaire means depending on the measure, medians, standard deviations), Cronbach’s alphas, and Spearman’s correlation coefficients were calculated for continuous variables. Test–retest reliability was assessed with intraclass correlation coefficient with two-way mixed model and absolute agreement in samples from the United Kingdom, Spain, and Hungary. MHS-R levels as a function of dichotomous and categorical demographic variables were tested using Mann–Whitney *U* tests and Kruskal–Wallis *H* tests, respectively (as the data did not fulfill the criteria to use their parametric counterparts). Dunn’s pairwise tests with the Bonferroni correction were carried out for the post-hoc tests in the case of the Kruskal–Wallis tests. Effect sizes *r* were calculated for the Mann–Whitney tests (*r* = Z/√*N*). According to Cohen’s (1988) classification of effect sizes, *r* from 0.10 to 0.29 means a small effect, from 0.30 to 0.49 a moderate effect, and above 0.50 a large or strong effect. In the case of the Kruskal–Wallis tests, eta squared was calculated, η2 = (H − *k* + 1)/(*n* − *k*). The benchmarks that define small, medium, and large effect sizes are η2 = 0.01, η2 = 0.06, and η2 = 0.14, respectively (Cohen, 1988). To evaluate the effects of MHS-R on other variables, a series of hierarchical linear regression analyses were employed, in each case, controlling for the effect of the country in the first step.

## Results

### Mature Happiness Scale-Revised

We conducted EFA on the first random half of the Canadian sample (*n* = 201). Data were suited for factor analysis, as the Kaiser–Meyer–Olkin measure of sampling adequacy was 0.90. Bartlett’s test of sphericity was significant, χ^2^ = 1086.35, *df* = 66, *p* < 0.001.

A one-factor solution explained 46.91% of the variance. According to parallel analysis (Hayton et al., 2004), the eigenvalue of the second possible factor (1.27) remained below the established threshold (1.37) calculated for this specific case, thus it was not retained. The three negatively-worded items showed the lowest factor loadings, warranting their removal (factor loadings of 0.07, − 0.11, and 0.42). The remaining factor loadings were as follows: 0.69, 0.50, 56, 68, 0.81, 0.89, 0.80, 0.70, 0.68.

This nine-item solution then was assessed with CFA on the second random half of the Canadian sample (*n* = 189). The fit was not satisfactory, χ2 = 84.54, *df* = 27, *p* < 0.001, CFI = 0.912, TLI = 0.883, RMSEA = 0.106 [90% CI 0.081, 0.132], SRMR = 0.052. Modification indices, residual variances, and regression weights were assessed, and two items were removed to make the model more parsimonious (“I have learned to be content in every situation.” and “I am able to give thanks at all times.”). Fit of the final, seven-item solution was excellent, χ2 = 13.00, *df* = 14, *p* = 0.526, CFI = 1.00, TLI = 1.00, RMSEA = 0.00 [90% CI 0.00, 0.066], SRMR = 0.024. The same one-factor structure was deemed to be an acceptable fit in all samples: United States, χ2 = 21.67, *df* = 14, *p* = 0.086, CFI = 0.987, TLI = 0.981, RMSEA = 0.050 [90% CI 0.00, 0.088], SRMR = 0.030, United Kingdom, χ2 = 58.85, *df* = 14, *p* < 0.001, CFI = 0.956, TLI = 0.934, RMSEA = 0.079 [90% CI 0.059, 0.101], SRMR = 0.039, Spain χ2 = 58.60, *df* = 14, *p* < 0.001, CFI = 0.972, TLI = 0.957, RMSEA = 0.066 [90% CI 0.049, 0.084], SRMR = 0.028, and Hungary, χ2 = 37.29, *df* = 14, *p* < 0.001, CFI = 0.962, TLI = . 944, RMSEA = 0.077 [90% CI 0.048, 0.107], SRMR = 0.035.

In all countries, item-total correlations ranged between 0.56 and 0.83 and regression weights ranged between 0.502 and 0.880 (see Table 2). The questionnaire also showed good internal consistency as Cronbach’s alphas in Canada, United States, United Kingdom, Spain, and Hungary were 0.87, 0.88, 0.85, 0.87, and 0.86, respectively. Moreover, kurtosis and skewness were in the acceptable range in all samples (see Table 3), although formal normality testing (Kolmogorov–Smirnov and Shapiro–Wilk tests) showed non-normal distribution in each country, *p* > 0.001. intraclass correlation coefficients were 0.83, 0.78 and 0.83 in the UK (*n* = 25), in Spain (*n* = 41), and in Hungary (*n* = 29), respectively, indicating good test–retest reliability of the measure. Detailed descriptive statistics of the MHS-R in all samples can be observed in Table 3.

**Table 2:** CFA standardized regression weights and standardized residual variances per country

	Canada (2^nd^ random half,* n* = 189)		United States (*n* = 223)		United Kingdom (*n* = 512)		Spain (*n* = 724)		Hungary (*n* = 281)	
	e	r. v	e	r. v	e	r. v	e	r. v	e	r. v
1. I am able to maintain inner peace	.793	.327	.880	.225	.812	.341	.758	.425	.773	.402
2. I feel comfortable in my own skin	.686	.529	.758	.426	.763	.418	.794	.370	.739	.453
3. I have learned to accept life as it is	.749	.439	.732	.465	.613	.625	.735	.460	.730	.467
4. I am at peace with myself	.861	.259	.856	.266	.879	.227	.829	.312	.843	.289
5. I have learned to remain calm, whatever comes	.675	.544	.649	.579	.566	.679	.703	.506	.622	.613
6. I am able to live in harmony with people that matter to me	.635	.596	.515	.734	.508	.742	.590	.652	.502	.748
7. I have learned to let go of all my cares and burdens	.620	.616	.697	.515	.561	.685	.532	.717	.626	.608

*e.* standardized estimate, *r. v.* standardized residual variance

**Table 3:** Descriptive statistics of the MHS-R in all countries

	Canada *(n* = 390)	United States (*n* = 223)	United Kingdom (*n* = 512)	Spain (*n* = 724)	Hungary (*n* = 281)	Total sample (*N* = 2130)
	*M (SD)*	*M (SD)*	*M (SD)*	*M (SD)*	*M (SD)*	*M (SD)*
*Age*						
18–34	23.79 (5.37)	22.68 (5.77)	21.55 (5.32)	24.43 (5.10)	22.98 (4.99)	23.44 (5.33)
35–44	24.46 (5.65)	25.08 (4.31)	22.98 (4.79)	24.26 (4.84)	23.43 (5.30)	24.02 (5.06)
45–84	26.12 (4.70)	26.04 (5.09)	23.31 (5.91)	25.09 (5.49)	25.43 (4.85)	24.77 (5.53)
*Gender*						
Female	24.30 (5.62)	24.56 (5.37)	22.59 (5.61)	24.24 (5.13)	23.61 (5.16)	23.74 (5.41)
Male	24.78 (5.07)	25.83 (5.03)	23.28 (5.30)	25.62 (5.07)	25.33 (4.58)	25.00 (5.10)
*Mental disorder diagnosis*						
Not diagnosed	25.09 (5.16)	25.82 (5.09)	23.76 (5.14)	25.11 (4.82)	24.23 (4.78)	24.75 (5.01)
Diagnosed	21.43 (5.49)	22.02 (4.98)	19.55 (5.61)	20.37 (5.63)	18.05 (6.05)	20.39 (5.60)
*Religiousness*						
Religious	25.39 (5.48)	25.64 (5.18)	23.68 (5.39)	24.45 (5.25)	23.92 (5.10)	24.57 (5.33)
Not religious	23.43 (5.02)	22.79 (5.17)	21.91 (5.60)	24.63 (5.08)	23.55 (5.20)	23.46 (5.34)
*Marital status*						
Single/divorced/widowed	23.23 (5.23)	24.72 (5.44)	22.10 (5.92)	24.38 (5.26)	23.04 (5.43)	23.46 (5.54)
In a relationship	24.65 (5.53)	23.33 (5.86)	21.92 (5.84)	24.65 (4.95)	24.88 (5.13)	23.99 (5.42)
Married/cohabiting with partner	25.27 (5.29)	25.16 (5.11)	23.47 (5.09)	24.65 (5.15)	23.99 (4.85)	24.47 (5.16)
*Highest level of education*						
Primary/secondary	22.83 (5.93)	26.06 (5.05)	22.23 (6.28)	23.32 (6.46)	23.29 (6.04)	23.06 (6.21)
Higher education	24.86 (5.28)	24.86 (5.22)	23.31 (5.32)	25.00 (4.71)	24.19 (4.73)	24.55 (5.03)
Still a student	24.62 (4.76)	23.27 (5.85)	21.85 (4.99)	23.46 (5.48)	21.52 (5.31)	22.84 (5.30)
*α*	.87	.88	.85	.87	.86	.87
Skewness (*SE*)	− 0.26 ( .12)	− 016 ( .16)	− 0.42 ( .11)	− 0.46 ( .10)	− 0.72 ( .15)	− 0.42 ( .53)
Kurtosis (*SE*)	− .41 ( .25)	− .28 ( .32)	− .15 ( .22)	− .07 ( .18)	.44 ( .18)	− .06 ( .11)

### Measurement Invariance

Configural, metric, and scalar invariance was tested among different countries, age groups, gender, and mental health diagnosis status. In all multi-group analyses, configural and metric invariance were held (see Table 4). In the case of age and mental disorder diagnosis, scalar invariance was also obtained. Regarding the country and gender, only scalar invariance was not supported, which means that item intercepts were not fully equal across groups. These results indicate that we can at least assume a quantitatively invariant measurement model of latent constructs across all tested groups, which supports the reliability of the measure.

**Table 4:** Invariance testing of MH-R (*N* = 2130)

Model	Comparison	χ2	*df*	Δχ2, scaled	Δ*df*	*p* for Δχ2	RMSEA	ΔRMSEA	CFI	ΔCFI	SRMR	ΔSRMR
*Country*												
1. Configural	–	211.20***	70	–	–	–	.069 [CI .058, .080]	–	.969	–	.032	–
2. Metric	1	269.31***	94	64.56	24	< .001	.066 [CI .057, .075]	.003	.962	.007	.063	.029
3. Scalar	2	390.35***	118	131.24	24	< .001	.074 [CI .066, .082]	.008	.941	.021	.076	.013
*Age*												
4. Configural	–	183.54***	42	–	–	–	.069 [CI .059, .079]	–	.968	–	.030	–
5. Metric	4	196.74***	54	8.56	12	.740	.061 [CI .052, .070]	.008	.968	.000	.037	.007
6. Scalar	5	232.83***	66	34.42	12	< .001	.060 [CI .051, .068]	.001	.962	.006	.042	.005
*Gender*												
7. Configural	–	153.59***	28	–	–	–	.065 [CI .055, .075]	–	.971	–	.028	–
8. Metric	7	167.00***	34	10.01	6	.124	.061 [CI .052, .070]	.004	.969	.002	.038	.010
9. Scalar	8	230.33***	40	72.84	6	< .001	.067 [CI .059, .075]	.006	.956	.014	.050	.012
*Mental disorder diagnosis*												
10. Configural	–	158.66***	28	–	–	–	.066 [CI .056, .076]	–	.967	–	.029	–
11. Metric	10	175.48***	34	12.80	6	.046	.063 [CI .054, .072]	.003	.965	.002	.038	.009
12. Scalar	11	198.98***	40	22.06	6	.001	.061 [CI .053, .070]	.002	.960	.005	.045	.007

Δ refers to change in the respective statistic. *p < .050; **p < .010; ***p < .001. Two-tailed

Country: Canada (*n* = 390); United States (*n* = 223); United Kingdom (*n* = 512); Spain (*n* = 724); Hungary (*n* = 281)

Gender: female (*n* = 1648); male (*n* = 482)

Age: 18–34 (*n* = 728); 35–44 (*n* = 731); 45–84 (*n* = 671)

Mental disorder diagnosis: no (*n* = 1774); yes (*n* = 356)

### MHS-R and Socio-demographic Data

In the entire sample, older participants tended to show higher MHS-R scores than younger ones (*Hypothesis 1*), *r*_*S*_ = 0.138, *p* < 0.001. Similarly, males (*Mdn* = 25), compared to females (*Mdn* = 24) showed higher scores on the measure, *U* = 350,619.00, *Z* =  − 3.93, *p* < 0.001, *r* = 0.08.

In line with *Hypothesis 3*, participants diagnosed with mental disorder (*Mdn* = 21) reported lower scores in the MHS-R compared to those without such diagnosis (*Mdn* = 25), *U* = 177,333.50, *Z* =  − 13.10, *p* < 0.001, *r* = 0.28. Religious participants (*Mdn* = 25) had higher score on this questionnaire than non-religious ones (*Mdn* = 24), *U* = 1,051,885.00, *Z* =  − 4.91, *p* < 0.001, *r* = 0.11.

Marital status, *H*(2) = 15.53, *p* < 0.001, η2 = 0.006, education level, *H*(2) = 39.80, *p* < 0.001, η2 = 0.018, and economic status, *H*(2) = 42.10, *p* < 0.001, η2 = 0.019, were related to scores obtained on MHS-R. Post-hoc Dunn’s pairwise tests with Bonferroni corrections showed that married respondents (*Mdn* = 25) indicated higher levels of mature happiness than their single counterparts (*Mdn* = 24), *Z* = 3.93, *p* < 0.001, *r* = 0.08. While participants in non-cohabiting relationships did not differ significantly from the other two groups, *p* > 0.05. Respondents with average economic status (*Mdn* = 25) showed higher scores on mature happiness than participants with higher than average (*Mdn* = 24) and lower than average levels (*Mdn* = 23) (Below average-average: *Z* = 4.34, *p* < 0.001, *r* = 0.09, below average-above average: *Z* = 6.49, *p* < 0.001, *r* = 0.14, average-above average: *Z* = 3.81, *p* < 0.001, *r* = 0.08. Other comparisons were not significant, *p* > 0.05. The same trends can be observed in each country separately (see Table 3).

### Correlates and Predictive Power of the MHS-R

Table 5 shows the average data of the study questionnaires of the study in all participating countries. As can be seen in Table 6, MHS-R scores positively correlated with all positive subscales of the PERMA-Profiler, namely with physical health and well-being. In line with *Hypothesis 2*, strong correlations were found between MHS-R scores and positive emotion (*r*_*S*_ = 0.71), meaning (*r*_*S*_ = 0.61) and general well-being (*r*_*S*_ = 0.69). We also observed a strong negative correlation with the negative emotion subscale (*Hypothesis 3, r*_*S*_ =  − 0.53) and a moderate correlation with the loneliness subscale (*r*_*S*_ =  − 0.36) of the PERMA-Profiler. It also showed a moderate inverse association with inner conflict (*r*_*S*_ =  − 0.49) and a strong negative association with the AAQ-II (*Hypothesis 4, r*_*S*_ =  − 0.59). All correlations were significant at the *p* < 0.001 level (see Table 6). Inner conflict was also strongly positively associated with both AAQ-II, *r*_*S*_ = 0.65, *p* > 0.001, and negative emotion, *r*_*S*_ = 0.50, *p* > 0.001.

**Table 5:** Descriptive statistics across variables

	Canada	United States	United Kingdom	Spain	Hungary	Total
*PERMA-Profiler*						
Positive emotion *M (SD)*	11.39 (3.67)	11.79 (3.55)	10.52 (4.01)	11.92 (3.62)	11.98 (3.27)	11.48 (3.72)
Engagement *M (SD)*	12.01 (3.36)	12.56 (3.45)	11.62 (4.03)	12.89 (3.42)	12.91 (2.89)	12.39 (3.54)
Relationship *M (SD)*	12.49 (3.79)	13.17 (3.75)	12.21 (4.36)	13.62 (3.63)	13.76 (3.97)	13.04 (3.95)
Meaning *M (SD)*	12.47 (3.92)	13.84 (3.37)	11.74 (4.49)	13.17 (4.15)	13.78 (3.62)	12.85 (4.12)
Accomplishment *M (SD)*	11.83 (3.16)	12.58 (3.36)	10.99 (3.86)	11.36 (3.74)	13.15 (2.90)	11.72 (3.60)
Wellbeing *M (SD)*	64.28 (16.24)	68.22 (15.44)	60.89 (18.92)	67.18 (17.07)	69.74 (13.98)	65.58 (17.12)
Negative emotion *M (SD)*	7.90 (3.80)	7.57 (3.90)	9.04 (3.98)	7.18 (3.71)	8.05 (3.54)	7.91 (3.85)
Health *M (SD)*	12.00 (3.60)	12.21 (3.80)	10.83 (4.22)	12.42 (3.6)	12.82 (3.48)	11.99 (3.84)
Loneliness *M (SD)*	2.68 (1.85)	2.50 (1.91)	2.70 (2.03)	2.08 (1.83)	2.35 (2.00)	2.42 (1.93)
Psychological inflexibility *M (SD)*	21.64 (9.67)	18.68 (9.00)	23.12 (11.03)	19.85 (9.70)	20.55 (10.20)	20.93 (10.13)
Inner conflict *M (SD)*	4.93 (1.98)	4.37 (1.86)	5.20 (2.15)	4.72 (1.96)	4.67 (1.93)	4.83 (2.01)
Mature happiness *M (SD)*	24.53 (5.37)	24.79 (5.32)	22.67 (5.57)	24.56 (5.14)	23.79 (5.13)	24.02 (5.36)

*M* represents questionnaire means

**Table 6:** Correlations between mature happiness (MHS-R) and other measures of the study using *rho*

Measure	Canada *(n* = 390)	United States (*n* = 223)	United Kingdom (*n* = 512)	Spain (*n* = 724)	Hungary (*n* = 281)	Total sample (*N* = 2130)
*PERMA-Profiler*						
Positive emotion	.74***	.69***	.70***	.69***	.73***	.71***
Engagement	.48***	.49***	.55***	.48***	.29***	.48***
Relationship	.56***	.41***	.47***	.50***	.23***	.46***
Meaning	.64***	.59***	.63***	.59***	.55***	.61***
Accomplishment	.55***	.56***	.56***	.56***	.54***	.55***
Well-being	.72***	.67***	.71***	.68***	.63***	.69***
Negative emotion	− .54***	− .61***	.56***	− .42***	− .56***	− .53***
Health	.48***	.38***	.40***	.39***	.28***	.41***
Loneliness	− .47***	− .36***	− .37***	− .25***	− .42***	− .36***
Psychological inflexibility	− .54***	− .61***	− .66***	− .54***	− .63***	− .59***
Inner conflict	− .44***	− .48***	− .54***	− .43***	− .57***	− .49***

*p < .050; **p < .010; ***p < .001. Two-tailed

After controlling for the effect of country, MHS-R scores explained 50% of the variance of well-being of PERMA, β = 0.70, *p* < 0.001. In separate analyses, it explained 19.1% of the variance of physical health, β = 0.44, *p* < 0.001; and 36.4% of the variance of AAQ-II, β = − 0.60, *p* < 0.001.

## Discussion

This study aimed to evaluate the psychometric properties of the MHS (Wong & Bowers, 2018), a recently proposed measure of mature happiness. For that purpose, we employed the original MHS, the PERMA-Profiler, and the AAQ-II in a cross-cultural sample including participants from Canada, the United States, the United Kingdom, Spain, and Hungary.

### Factor Structure and Internal Consistency

In terms of factor structure, the final model, providing an excellent fit to the data in all participating countries, was the one-factor solution with seven items. To obtain this fit, reversed items from the original measure were removed, suggesting that the items measuring inner conflict may be separate from the construct of mature happiness. Similarly, the only item referring to gratitude showed to be not part of the MHS-R, which means that gratitude and mature happiness may be different, albeit quite possibly related concepts. We called the resulting scale the Mature Happiness Scale-Revised (MHS-R). Configural and metric measurement invariance of this scale were observed across countries, age groups, gender, and mental disorder diagnosis, supporting the notion that the psychological meanings measured by the MHS-R were equivalent across all tested groups. Cronbach’s alphas were high in all five countries (ranging from 0.85 to 0.88), as was the test–retest reliability after a period of 3 to 4 weeks (intraclass correlation coefficients of 0.75 and 0.83).

Overall, these findings show a solid structure and reliability of the MHS-R in all five cultures, in three different languages. As this is the first study evaluating the factor structure and internal consistency of the MHS, we recommend the use of our revised version in future studies. These results also provide evidence of a priori incremental validity of the MHS-R in comparison with the Peace of Mind Scale (Lee et al., 2013) and the Harmony in Life Scale (Kjell & Diener, 2021) since it has shown measurement invariance across more groups than the latter scales (across age groups, clinical/non-clinical sample, and five countries). These findings support the meaningfulness of the comparisons of the MHS-R scores between these populations.

### Validity Evidence Based on Relations with Other Variables

To evaluate the validity of the MHS-R based on relations with other variables, we formulated four hypotheses. *Hypothesis 1* predicted that mature happiness would increase across the lifespan. This hypothesis was supported since older people showed higher scores in mature happiness than younger ones, albeit the effect size was small. This may be because mature happiness requires high levels of emotional maturity and regulation, which typically increases with age (e.g., Lawton et al., 1992; Márquez-González et al., 2008). The findings are consistent with previous studies showing that well-being increases across the lifespan in Western countries (e.g., Schönfeld et al., 2017), similarly to meaning in life (Carreno et al., 2020; Schnell, 2009; Steger et al., 2009) At the same time, levels of mental illness typically decrease (e.g., Westerhof & Keyes, 2010). In addition, males scored higher in mature happiness than females. In the literature, there seems to be a controversy about the possible gender-based differences in well-being, thus this finding requires further studies (e.g., Batz-Barbarich et al., 2018; Schönfeld et al., 2017).

In line with *Hypothesis 2*, mature happiness was strongly associated with the PERMA-Profiler scores in physical health and well-being, including the subscales of positive emotions, engagement, relationships, meaning, and accomplishment. Mature happiness explained up to half of the variance of the PERMA well-being. These results provide evidence of the concurrent validity of the MHS-R as a measure of well-being. The strongest correlations were observed with positive emotions and meaning in life, demonstrating that mature happiness most probably includes aspects of both subjective and eudaimonic well-being (Carreno et al., 2021).

Despite these positive associations, there is empirical evidence showing that mature happiness represents a different construct from the PERMA-Profiler (Carreno et al., 2021). Mature happiness is a positive state based on inner harmony and peace of mind as a result of living in balance between the positive and negative aspects of one’s life. Other traditional measures of well-being such as the PERMA-Profiler (Butler & Kern, 2016), the Oxford Happiness Questionnaire (Hills & Argyle, 2002), or the Satisfaction with Life Scale (Diener et al., 1985) do not include these harmony-based features as they exclusively focus on subjective and eudaimonic well-being. Indeed, recent findings have shown that the PERMA-Profiler and Diener’s (1984) model of subjective well-being do not represent different factors but the same well-being factor (Goodman et al., 2018). This is not the case of mature happiness (see Carreno et al., 2021), which supports the idea that mature happiness may represent a third type of happiness called chaironic happiness (Wong, 2011).

As predicted by *Hypothesis 3*, mature happiness was negatively related to negative affect and inner conflict. In the case of negative affect, this association was stronger than predicted. We observed that people diagnosed with a mental health disorder scored significantly lower in mature happiness than people without such diagnosis (although with a small effect). In general, these findings show the discriminant validity of the MHS-R for the prediction of mental health problems. Based on the inclusion of negative aspects of living into the conceptualization of mature happiness (Wong, 2020; Wong & Bowers, 2018), the MHS-R seems to be a measure of well-being with more predictive power of distress than other well-being measures. For instance, Carreno et al. (2021) observed that the MHS-R showed a better predictive power of psychological distress (particularly of stress and anxiety) during the COVID-19 pandemic than the PERMA-Profiler. Together with the measurement invariance between clinical and non-clinical samples, the evidence collected in this paper encourages the reliable use of the MHS-R in studies and interventions including people with mental health problems.

Moreover, mature happiness was negatively associated with loneliness, a factor linked to mental illness (Hawkley & Cacioppo, 2010; Mushtaq et al., 2014; Wang et al., 2018). The conceptualization of mature happiness highlights the relational facet of well-being (Wong, 2020; Wong & Bowers, 2018), being explicitly represented in one of the MHS-R items (“I am able to live in harmony with people that matter to me”). These results manifest the importance of social relatedness in the achievement of inner harmony and happiness in general, as previously suggested (e.g., Delle Fave et al., 2016; Kjell et al., 2016; Seligman, 2011).

Finally, we observed a strong negative relationship between mature happiness and psychological inflexibility, confirming our *Hypothesis 4*. Participants with higher scores on mature happiness reported a lower tendency to avoid negative emotions and thoughts. To the best of our knowledge, this is the first empirical study showing a connection between inner harmony and psychological flexibility, which thus far had been only theorized (Kjell et al., 2016; Wong, 2020). This finding supports the idea that mature happiness may be considered the experiential component of psychological flexibility. Putting it differently, to achieve mature happiness, most probably it is necessary to be psychologically flexible. The results also suggest that psychological inflexibility, understood as the rigid avoidance of negative emotions and thoughts (Bond et al., 2011), may represent an opposite state to mature happiness, the latter being characterized by acceptance (Wong & Bowers, 2018).

## Limitations and Conclusion

The main limitations of this study include a convenience sampling method, meaning that the distribution of the sample based on gender, age, educational level and other demographic data is not representative of the general population. For instance, our sample contains a high percentage of women, married individuals, and people with higher levels of education. As it is a cross-sectional study based on self-reported data, causal relationships cannot be confirmed, and it is possible that the findings were affected by the common method bias. The order of the questionnaires might have also been a source of bias.

Moreover, there was a missing criterion measure of harmony (e.g., the Peace of Mind Scale, Lee et al., 2013, or the Harmony in Life Scale, Kjell et al., 2016) to test convergent validity of the MHS-R as a measure of inner harmony. This happened because the original data collection belongs to a larger research project and an additional measure would have made the survey excessively long. Future studies could analyze this validity aspect of the MHS-R. Similarly, in order to understand more in depth the role of mature happiness in well-being, future studies are needed that compare this measure with other well-established measures of happiness or subjective well-being (e.g., Diener et al., 1985; Hills & Argyle, 2002). All these limitations are, however, at least partially mitigated by the multinational nature of the study and the fact that we mainly had participants from the community sample and not only undergraduate students.

In summary, this paper provides several indices of validity evidence of the MHS-R to measure mature happiness across five different countries in three languages. Mature happiness represents an empirically supported conceptualization of harmony-based well-being. The MHS-R, as a validated measure, can be a noteworthy contribution that opens the door for future empirical research on mature happiness across different populations and cultures.

## Data Availability

The raw data supporting the conclusions of this article will be made available by the authors, without undue reservation.

## Appendix: Mature Happiness Scale-Revised in English, Spanish (*S*), and Hungarian (*H*).

Considering your life overall, please characterize your life by rating the following statements using the scale (from 1 to 5) below.

*(S) Considerando su vida en general, por favor, caracterice su vida valorando las siguientes afirmaciones según la siguiente escala (1–5).*

*(H) Általánosságban szemlélve, kérjük jellemezze az életét a megadott állítások segítségével egy 1-től 5-ig terjedő skálán.*

**Table:**

1	2	3	4	5
Not at all *(S) Nada* *(H) Egyátalán nem*				All of the time *(S) Todo el tiempo* *(H) Mindig*
1. I am able to maintain inner peace *(S) Soy capaz de mantener paz interior* *(H) Képes vagyok megőrizni a belső békémet*				$$\begin{array}{*{20}l} 1 \hfill & 2 \hfill & 3 \hfill & 4 \hfill & 5 \hfill \\ \end{array}$$
2. I feel comfortable in my own skin *(S) Me siento cómodo/a en mi propia piel* *(H) Jól érzem magam a bőrömben*				$$\begin{array}{*{20}l} 1 \hfill & 2 \hfill & 3 \hfill & 4 \hfill & 5 \hfill \\ \end{array}$$
3. I have learned to accept life as it is *(S) He aprendido a aceptar la vida tal cual es* *(H) Megtanultam elfogadni az életet úgy ahogy van*				$$\begin{array}{*{20}l} 1 \hfill & 2 \hfill & 3 \hfill & 4 \hfill & 5 \hfill \\ \end{array}$$
4. I am at peace with myself *(S) Estoy en paz conmigo mismo/a* *(H) Békében vagyok önmagammal*				$$\begin{array}{*{20}l} 1 \hfill & 2 \hfill & 3 \hfill & 4 \hfill & 5 \hfill \\ \end{array}$$
5. I have learned to remain calm, whatever comes *(S) He aprendido a mantener la calma, venga lo que venga* *(H) Megtanultam, hogy nyugodt maradjak, bármi is történik*				$$\begin{array}{*{20}l} 1 \hfill & 2 \hfill & 3 \hfill & 4 \hfill & 5 \hfill \\ \end{array}$$
6. I am able to live in harmony with people that matter to me *(S) Soy capaz de vivir en armonía con la gente que me importa* *(H) Képes vagyok harmóniában élni azokkal, akik fontosak számomra*				$$\begin{array}{*{20}l} 1 \hfill & 2 \hfill & 3 \hfill & 4 \hfill & 5 \hfill \\ \end{array}$$
7. I have learned to let go of all my cares and burdens *(S) He aprendido a dejar de lado todas mis preocupaciones y cargas* *(H) Megtanultam elengedni minden gondomat és terhemet*				$$\begin{array}{*{20}l} 1 \hfill & 2 \hfill & 3 \hfill & 4 \hfill & 5 \hfill \\ \end{array}$$

## Code availability

Code is available upon request from the corresponding author.
